# Morphological indicators of puberty in 3/4 and 5/8 Girolando heifers

**DOI:** 10.1007/s11250-026-05078-7

**Published:** 2026-05-20

**Authors:** Marcelo José Böck, Ana Paula Pereira Schimidt, Jurandy Mauro Penitente-Filho, Mateus Guimarães dos Santos, Renata de Fátima Bretanha Rocha, José Domingos Guimarães, Simone Eliza Facioni Guimarães

**Affiliations:** 1 Department of Animal Science, Universidade Federal de Viçosa, Viçosa, MG 36570-900 Brazil; 2 Department of Veterinary, Universidade Federal de Viçosa, Viçosa, MG 36570-900 Brazil; 3 JPF Consultoria, Santa Teresa, ES 29650-000 Brazil

**Keywords:** Body measures, Dairy cattle, Genetic groups, Oulation, Survival rate

## Abstract

Age at puberty is a key determinant of reproductive efficiency in dairy systems and may vary according to genetic composition and body development. The aim of this study was to evaluate morphometric predictors of puberty onset and to compare puberty timing between 3/4 and 5/8 Girolando heifers. A total of 60 heifers (3/4, *n* = 18; 5/8, *n* = 42), aged 6.9 to 13.5 months, were raised under semi-intensive system. Every 21 days, morphometric traits were recorded, and ovarian ultrasonography was performed to detect the presence of corpus luteum (CL). Linear regression was used to describe age-related changes in morphometric traits, logistic regression to estimate the probability of ovulation, and survival analysis (Cox regression) to evaluate time to puberty. Morphometric traits increased significantly with age in both genetic groups. Rump length, ilium width, rump area, and estimated body weight were associated with a 50% probability of ovulation at 45.30 cm, 40.87 cm, 1506.91 cm², and 339.06 kg, respectively. Mean age at puberty was 452.8 ± 20.18 days for 3/4 and 481.14 ± 28.99 days for 5/8 heifers (*p* > 0.4). Survival analysis indicated that the probability of not reaching puberty began to decline at approximately 55 weeks of age. Rump area was the morphometric trait most strongly associated with earlier ovulation. In conclusion, pelvic morphometric traits, particularly rump area adjusted to 365 days of age, are relevant indicators of reproductive maturation in Girolando heifers and may support management decisions aimed at improving reproductive efficiency.

**Supplementary Information:**

The online version contains supplementary material available at 10.1007/s11250-026-05078-7.

## Introduction

The growing demand for young and productive dairy cattle intensified efforts to optimize reproductive management, aiming at greater efficiency in production systems. The early entry of heifers into the system helps to increase both the quantity and quality of milk produced, making dairy production more sustainable and economically viable (De Vries 2020). However, this pursuit of higher reproductive precocity presents considerable challenges, one of which is the increased incidence of parturition complications. These complications are frequently associated with inappropriate use of breeds in crossbreeding systems, leading to early animal culling and compromising the longevity of heifers in the herd (Overton and Dhuyvetter 2020). At the same time, the undervaluation of calves and heifers within production systems aggravates this problem, as these animals are often viewed merely as sources of immediate cost rather than strategic investments in the future profitability. This situation negatively affects the farm performance indices, impairing production planning and long-term sustainability (Boulton et al. 2017).

Early puberty is a crucial factor for reproductive success, as it influences skeletal development and pelvic formation, essential structures for pregnancy and lactation, allowing heifers to enter the production cycle earlier and accelerating the return on investment (Duittoz et al. 2016). Nevertheless, the age at puberty can vary substantially among different genetic compositions, especially in crossbreeding systems in which productive and adaptive traits are combined in different ways (Cousminer et al. 2016; Zhu et al. 2018). Morphometric measurements are widely used to assess and predict the onset of puberty. Traits such as height, length, and thoracic circumference provide indicators of developmental progress, as demonstrated in studies involving cattle (Fernandes et al. 2022), goats (Fonseca et al. 2021), and sheep (Silva et al. 2019). These measurements therefore represent practical tools for estimating physical maturity and linking growth to reproductive readiness.

Pelvic morphology, including rump area and related measurements, may represent an integrative outcome of growth and endocrine activity. Estrogen plays a central role in bone development and pelvic growth during puberty, influencing epiphyseal closure and skeletal shape (Nilsson et al. 2001). Evidence from developmental biology indicates that pelvic sexual dimorphism emerges early and is regulated by differential activation of sex hormone receptors throughout growth (Iguchi et al. 1989; Uesugi et al. 1992; Kanahashi et al. 2024). Although direct associations between pelvic morphometry and puberty onset in cattle remain limited, recent findings in Gyr cattle demonstrated moderate genetic correlations between pelvic linear traits, particularly ilium width, rump area, and hip height, and reproductive outcomes such as in vitro embryo production (Machado et al. 2024), supporting the hypothesis that these traits may serve as indirect indicators of reproductive maturation.

The selection of breed and genetic composition plays a fundamental role in productive and reproductive performance, especially in crossbreeding systems. The Girolando breed, resulting from crosses between Gyr and Holstein cattle, is one of the most representative dairy cattle in Brazil and is known for superior fertility and survival (Heins et al. 2006) as well as resistance to tropical environmental conditions (da Costa et al. 2020). Despite its importance, limited information is available regarding differences in morphometric development and puberty timing between the 3/4 and 5/8 genetic groups. It is hypothesized that these groups exhibit distinct morphometric profiles and puberty onset patterns. Therefore, this study aimed to compare morphometric measures to identify and distinguish puberty timing between 3/4 and 5/8 Girolando heifers.

## Material and methods

### Ethics and animals

The Animal Use Ethics Committee of the Universidade Federal de Viçosa (CEUA-UFV) approved the implementation of this project (Protocol no. 51/2019).

This study was conducted with 60 Girolando heifers from two genetic groups (3/4 GG, n = 18 and 5/8 GG, n = 42) raised under a semi-intensive system on a commercial farm located in Minas Gerais, Brazil (21° 21’ 33.9” S, 43° 01’ 57.5” W). The climate of the region is classified as tropical, with an average annual temperature of approximately 21.2 °C, and an average altitude of 440 m above sea level.

At the beginning of the experiment, heifers aged between 6.9 and 13.5 months and were maintained in paddocks with Tifton grass. Animals were supplemented with corn silage, soybean meal, cornmeal, and mineral salt, targeting an expected average daily gain of 670 g.d^− 1^.

### Rectal palpation and ultrasonography

The reproductive tract status and ovarian activity were assessed every 21 days by rectal palpation followed by transrectal ultrasonography. Each heifer was evaluated between seven and 15 times throughout the experimental period. Ultrasonography was performed using a B-mode ultrasound device equipped with a 7.5 MHz transrectal electronic transducer (Mindray^®^, model DP 2200 Vet, Mainland, China) to identify and assess uterus and ovaries and to visually monitor their development, verifying the presence, number, and size of ovarian follicles and corpus luteum.

### Morphometric traits

**Fig. 1:**
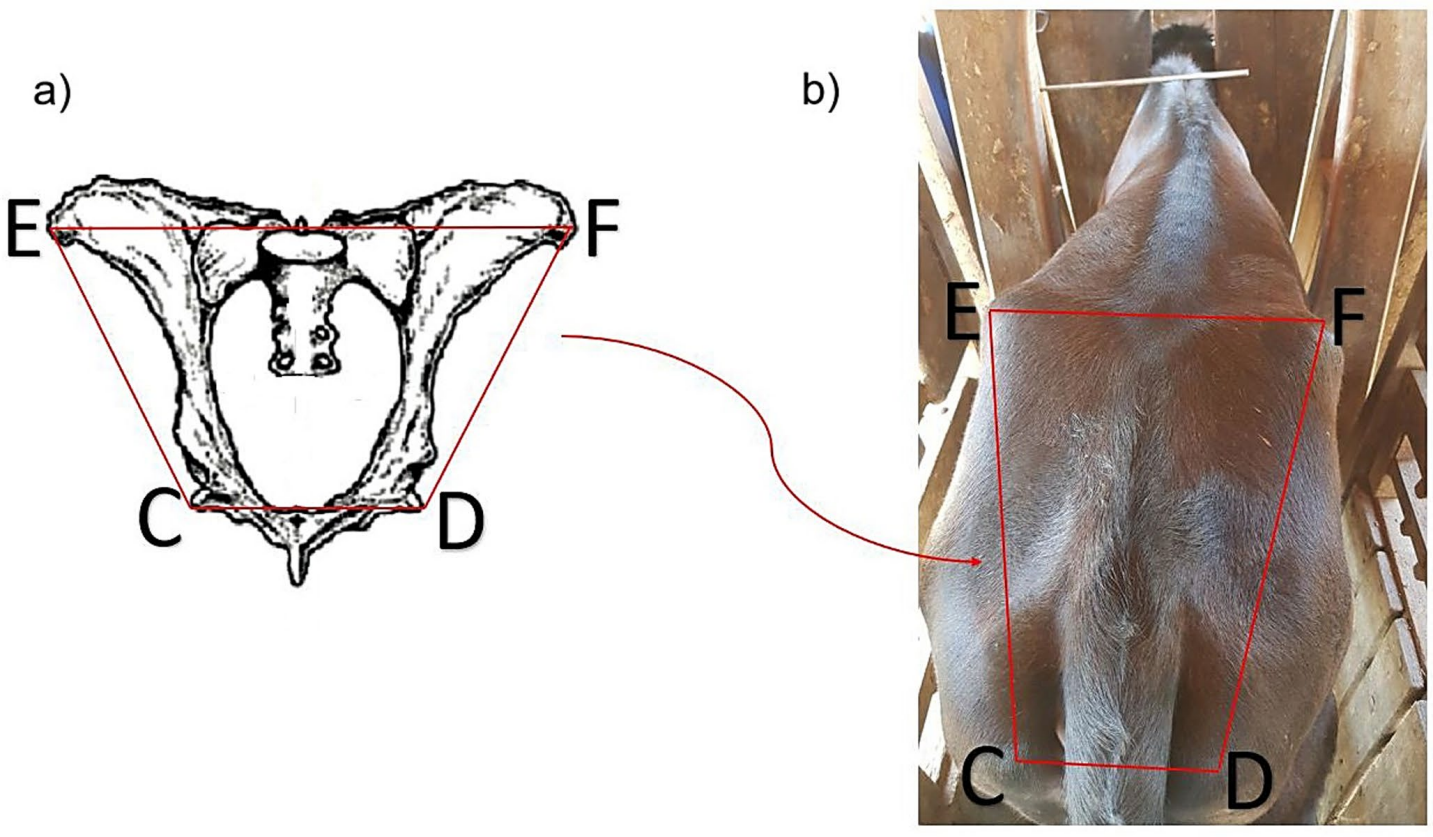
Representative drawing of the bovine pelvic bone structure as a trapezoid (**a**) and the bovine rump (**b**). Letters **E** and **F** indicate left and right iliac tuberosities, respectively, and the distance between them represents ilium width. Letters **C** and **D** indicate left and right ischial tuberosities, respectively, and the distance between them represents ischium width. The distances between **E** and **C** and between **F** and **D** represents rump length

Morphometric traits were assessed every 21 days. Due to the absence of scales on the farm, estimated body weight was obtained using a weight estimation tape positioned around the thoracic perimeter, according to standardized values ​​for medium-sized breeds. The withers height was measured using a hypometer, recording the distance from the ground to the dorsal end of the spinous processes of the first thoracic vertebrae. The rump length (distance between the ilium and ischium), ilium width (distance between the left and the right iliac tuberosities), and ischium width (distance between the left and right ischial tuberosities) were measured using an adapted graduated ruler. Based on these pelvic measurements, the rump area was calculated in cm^2^. Considering the pelvis to approximate the shape of an isosceles trapezoid (Fig. 1), the following formula was used to calculate the area:

$$\:\mathrm{A}=\frac{\left(\mathrm{B}+\mathrm{b}\right)\mathrm{*}\mathrm{h}}{2}$$

Where $\:\mathrm{A}$ is the area of ​​the trapezium/rump; $\:\mathrm{B}$ is the larger base (ilium width), $\:\mathrm{b}$ is the smaller base (ischium width) and $\:\mathrm{h}$ is the trapezium height. The $\:\mathrm{h}$ measurement was obtained using the following formula:

$$\>{\rm{h}} = \sqrt {{{\rm{L}}^2}} - \left[ {{{{\rm{B}} - {{\rm{b}}^2}} \over 2}} \right]$$

where $\:\mathrm{L}$ is the distance between ilium and ischium (rump length).

### Statistical analysis

Morphometric traits were analyzed using linear mixed-effects models (Mixed Procedure, SAS OnDemand) according to age (in days) and genetic group (GG). Age was treated as a continuous covariate, and animal identification was included as a random effect to account for the correlation of repeated measurements within the same heifer. The model was specified as:

$$\:{\mathrm{Y}}_{\mathrm{i}\mathrm{j}}={{\upbeta\:}}_{0}+{{\upbeta\:}}_{1}{\mathrm{A}\mathrm{g}\mathrm{e}}_{\mathrm{i}\mathrm{j}}+{{\upbeta\:}}_{2}{\mathrm{G}\mathrm{G}}_{\mathrm{j}}+{\mathrm{u}}_{0\mathrm{j}}+{\mathrm{u}}_{1\mathrm{j}}{\mathrm{A}\mathrm{g}\mathrm{e}}_{\mathrm{i}\mathrm{j}}+{{\upepsilon\:}}_{\mathrm{i}\mathrm{j}}$$

Where $\:{\mathrm{Y}}_{\mathrm{i}\mathrm{j}}$ is the morphometric trait measured in animal $\:\mathrm{j}$ at measurement occasion $\:\mathrm{i}$; $\:{{\upbeta\:}}_{0}$ is the overall fixed intercept; $\:{{\upbeta\:}}_{1}$ is the fixed slope associated with age; $\:{\mathrm{A}\mathrm{g}\mathrm{e}}_{\mathrm{i}\mathrm{j}}$ is the age of animal $\:\mathrm{j}$ at measurement $\:\mathrm{i}$ (in days); $\:{{\upbeta\:}}_{2}$ represents the estimated difference between genetic groups (relative to the reference group), holding age constant; $\:{\mathrm{G}\mathrm{G}}_{\mathrm{j}}$ is the genetic group of animal $\:\mathrm{j}$ and treated as a categorical fixed effect; $\:{\mathrm{u}}_{0\mathrm{j}}$ and $\:{\mathrm{u}}_{1\mathrm{j}}$ are the random intercept and random slope for age associated with animal $\:\mathrm{j}$, respectively, $\:\left(\genfrac{}{}{0pt}{}{{\mathrm{u}}_{0\mathrm{j}}}{{\mathrm{u}}_{1\mathrm{j}}}\right)\sim\:\mathrm{N}\left(\left(\genfrac{}{}{0pt}{}{0}{0}\right),{{\Sigma\:}}_{\mathrm{u}}\right)$; and $\:{{\upepsilon\:}}_{\mathrm{i}\mathrm{j}}$ is the residual error term, $\:{{\upepsilon\:}}_{\mathrm{i}\mathrm{j}}\sim\:\mathrm{N}(0,{{\upsigma\:}}^{2})$.

In addition, individual growth curves were fitted (Reg Procedure) and used to adjust predicted values of morphometric traits at 365 days of age. Pearson correlation coefficients were calculated to assess the relationship between morphometric traits and age (Corr Procedure).

The effects of the 365-days-adjusted morphometric values on the probability of ovulation during the experimental period were analyzed by univariate logistic regression (Logistic Procedure). Probabilities were calculated by the formula (Hosmer et al. 2013).

$$\:\mathrm{P}=\frac{{\mathrm{e}}^{({{\upbeta\:}}_{0}+{{\upbeta\:}}_{1}{\mathrm{x}}_{1})}}{1+{\mathrm{e}}^{({{\upbeta\:}}_{0}+{{\upbeta\:}}_{1}{\mathrm{x}}_{1})}}$$

Where $\:\mathrm{P}$ represents the estimated probability of ovulation; $\:{{\upbeta\:}}_{0}$ is the intercept of the model; $\:{{\upbeta\:}}_{1}$ is the regression coefficient associated with the explanatory variable; and $\:{\mathrm{x}}_{1}$ corresponds to the 365-days–adjusted morphometric value included in the model.

The area under the ROC curve (AUC) was used to assess the discriminatory ability of each univariate logistic model, with values closer to 1.0 indicating greater discriminative capacity.

To facilitate biological interpretation, the values of the explanatory variables ($\:{\mathrm{x}}_{1}$) associated with a 50% estimated probability of ovulation were calculated from each univariate logistic regression model. This threshold represents the morphometric value at which the predicted probability of ovulation equals 0.50 and was obtained using the following equation:

$$\:{\mathrm{x}}_{1}=\frac{\mathrm{l}\mathrm{n}\left(\frac{0.5}{{\mathrm{e}}^{{{\upbeta\:}}_{0}}\left(1-0.5\right)}\right)}{{{\upbeta\:}}_{1}}$$

Subsequently, multivariate logistic regression was performed to identify the combination of morphometric and biological variables that best predicted the probability of ovulation during the experimental period. Initially, predictors were pre-selected using the Score chi-square statistic, ranking variables according to their univariate association with the outcome. Genetic group (GG) and body condition score (BCS) were retained based on their biological relevance. Multicollinearity among candidate predictors was evaluated using variance inflation factors (VIF) and Pearson correlation coefficients. VIF values greater than 10 or absolute correlation coefficients exceeding 0.80 were considered indicative of excessive collinearity; therefore, highly correlated variables were not included simultaneously in the same model. A forward selection was applied to the final model to estimate the Akaike’s information criterion corrected to small samples (AICc) in each step.

Survival analyses were conducted in R (version 4.3.3; R Core Team 2024), using the *survival*, *MuMIn*, *car*, *dplyr*, *tidyr*, *scales*, and *patchwork* packages. Cox proportional hazards regression models were used to analyze time-to-ovulation, defined as the age (in weeks) at which ovulation was first detected.

The initial multivariate Cox model included GG and BCS, and the following 365-days–adjusted morphometric traits: withers height, rump width, ilium width, ischium width, rump area, and body weight. The proportional hazards assumption was assessed using Schoenfeld residuals, and multicollinearity among covariates was assessed via VIF and Pearson correlation.

An automated model selection was performed using AICc, with GG and BCS retained in all candidate models and restrictions applied to prevent inclusion of highly correlated traits simultaneously. Model averaging was then used across the models to obtain robust estimates of covariate effects.

Based on model selection and averaging results, a final Cox proportional hazards model was constructed including GG, BCS, and rump area modeled as a penalized spline (*pspline*) to allow for potential non-linear effects. The proportional hazards assumption was re-evaluated and satisfied for the final model.

To evaluate the morphometric traits at the time of ovulation and compare them between the genetic groups, analysis of variance (GLM Procedure) was performed according to the model:

$$\:{\mathrm{Y}}_{\mathrm{i}\mathrm{j}}={\upmu\:}+{\mathrm{G}}_{\mathrm{i}}+{\mathrm{e}}_{\mathrm{i}\mathrm{j}}$$

Where: $\:{\mathrm{Y}}_{\mathrm{i}\mathrm{j}}$ is the value of the trait; $\:{\upmu\:}$ is the general constant; $\:{\mathrm{G}}_{\mathrm{i}}$ is the genetic group effect on the trait; and $\:{\mathrm{e}}_{\mathrm{i}\mathrm{j}}$ is the error.

Overall, the significant level adopted was α = 0.05, and tendency was considered when *p* < 0.10. All graphs were generated using *ggplot2* package in R environment.

## Results

Descriptive statistics of heifers’ body traits by age (in months) for 3/4 and 5/8 GG with averages and standard deviations are available in Suppl Table 1. Observed values of morphometric traits over time is in Suppl Fig. 1.

**Fig. 2:**
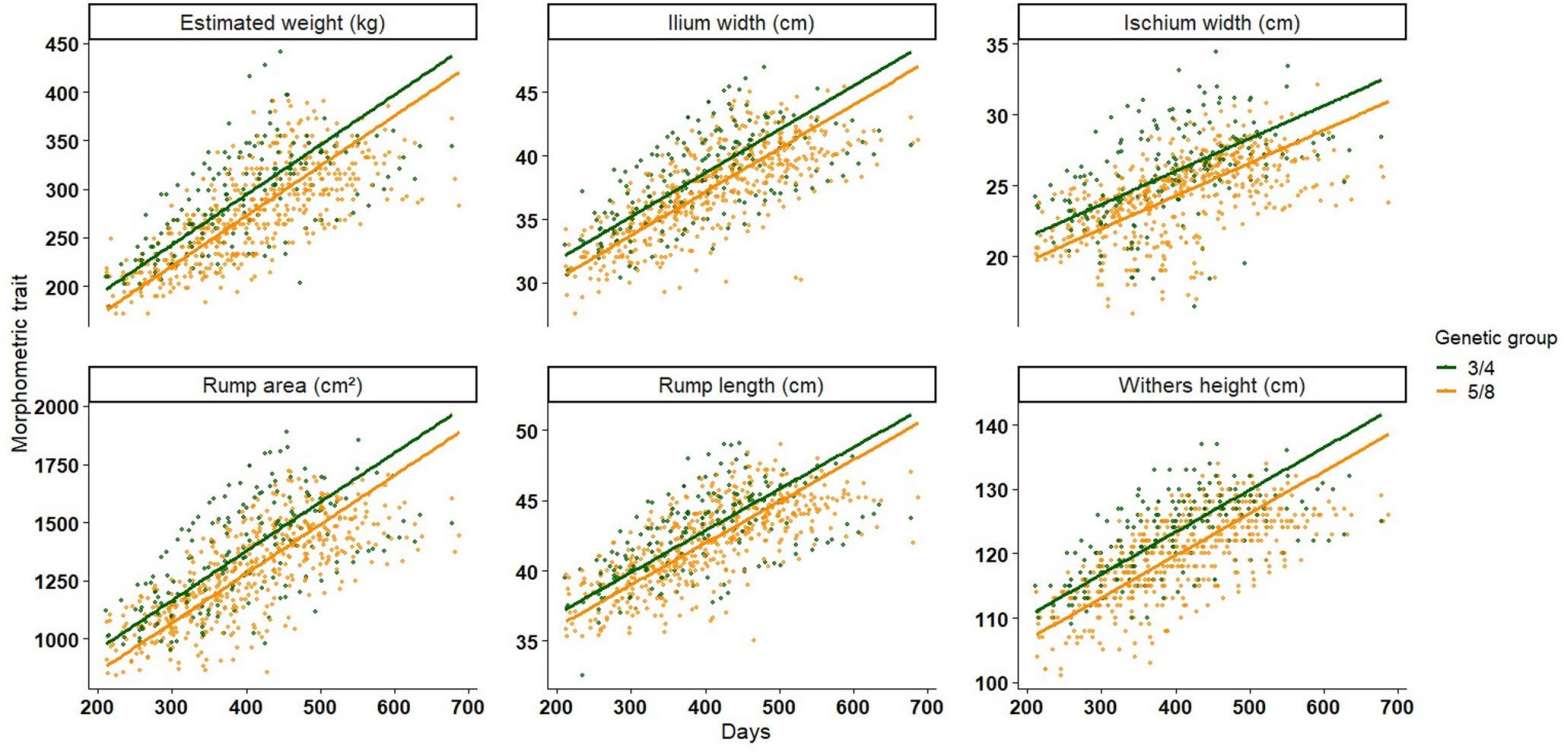
Values of morphometric traits over time according to genetic group in Girolando heifers

Age was highly significant for all evaluated morphometric traits (*p* < 0.0001; Table 1; Fig. 2). Withers height increased by an average of 0.07 ± 0.002 cm/day, while rump length and ilium width increased by 0.03 ± 0.001 cm/day and 0.04 ± 0.001 cm/day, respectively. Ischium width showed a slower but significant increase with age (0.02 ± 0.001 cm/day). Rump area expanded by 2.20 ± 0.06 cm²/day, and estimated body weight increased by 0.55 ± 0.018 kg/day.

The GG was significant (*p* < 0.05) for withers height and ischium width. Compared with 5/8 Girolando heifers, 3/4 heifers presented greater withers height (+ 2.88 ± 1.24 cm) and larger ischium width (+ 1.46 ± 0.49 cm). No significant GG differences were observed for rump length, ilium width, rump area, or estimated weight (*p* > 0.25).

**Table 1:** Estimate parameters (± SE) from the linear mixed-effects models for morphometric traits according to age and genetic group (GG) in Girolando heifers

Parameter	Estimate ± SE	*p*-value	95% LCL	95% UCL
*Withers height*				
Intercept	93.03 ± 0.821	< 0.0001	91.38	94.67
Age (d)	0.07 ± 0.002	< 0.0001	0.065	0.072
GG (3/4)	2.88 ± 1.241	0.0238	0.40	5.37
GG (5/8) ref.	0	-	-	-
*Rump length*				
Intercept	29.84 ± 0.32	< 0.0001	29.2	30.48
Age (d)	0.03 ± 0.001	< 0.0001	0.029	0.033
GG (3/4)	0.33 ± 0.488	0.5058	-0.65	1.30
GG (5/8) ref.	0	-	-	-
*Ilium width*				
Intercept	23.22 ± 0.361	< 0.0001	22.5	23.94
Age (d)	0.04 ± 0.001	< 0.0001	0.034	0.038
GG (3/4)	0.63 ± 0.559	0.2642	-0.49	1.75
GG (5/8) ref.	0	-	-	-
*Ischium width*				
Intercept	15.08 ± 0.371	< 0.0001	14.35	15.81
Age (d)	0.02 ± 0.001	< 0.0001	0.022	0.025
GG (3/4)	1.46 ± 0.493	0.0043	0.48	2.45
GG (5/8) ref.	0	-	-	-
*Rump area*				
Intercept	430.62 ± 18.898	< 0.0001	392.59	468.65
Age (d)	2.2 ± 0.06	< 0.0001	2.085	2.323
GG (3/4)	34.42 ± 29.692	0.2519	-25.23	94.08
GG (5/8) ref.	0	-	-	-
*Estimated weight*				
Intercept	62.14 ± 4.867	< 0.0001	52.37	71.9
Age (d)	0.55 ± 0.018	< 0.0001	0.512	0.583
GG (3/4)	5.82 ± 6.568	0.3798	-7.39	19.04
GG (5/8) ref.	0	-	-	-

Correlations between the age and all morphometric traits were moderate to high (Suppl Fig. 2).

Univariate Logistic Regression analyses indicated that several 365-day–adjusted morphometric traits were significantly associated with the probability of ovulation during the experimental period (Table 2). In these models, the regression coefficient (β) represents the change in the log-odds of ovulation per unit increase in the predictor.

Rump length showed a positive association with ovulation probability (β = 0.397 ± 0.181), corresponding to an odds ratio (OR) of 1.487. Thus, each additional centimeter in rump length at 365 days of age was associated with a 48.7% increase in the odds of ovulation. The rump length value associated with a predicted ovulation probability of 50% was 45.3 cm.

Similarly, ilium width was positively associated with ovulation probability (β = 0.369 ± 0.155; OR = 1.446), indicating a 44.6% increase in the odds of ovulation for each additional centimeter. The estimated ilium width corresponding to a 50% probability of ovulation was 40.9 cm.

Rump area also showed a significant association with ovulation probability (β = 0.006 ± 0.003). Although the effect per unit was small (OR = 1.006 per cm²), a more practical interpretation indicates that an increase of 100 cm² in rump area corresponds to an OR of 1.822, nearly doubling the odds of ovulation. The rump area associated with a 50% predicted probability of ovulation was 1506.9 cm².

Estimated body weight was positively associated with ovulation probability (β = 0.02 ± 0.01), with an OR of 1.02 per kilogram or 1.221 per 10 kg, indicating a 22.1% increase in the odds of ovulation for each additional 10 kg. A predicted ovulation probability of 50% was observed at an estimated body weight of 339.1 kg.

In contrast, withers height was not significantly associated with ovulation probability (*p* > 0.20). Ischium width showed a marginal association (*p* < 0.06) with ovulation probability (β = 0.334 ± 0.173, OR = 1.397).

**Table 2:** Parameter estimates ± SE of univariate logistic regression of 365d-adjusted morphometric traits on probability of ovulation in 3/4 and 5/8 Girolando heifers (*n* = 60)

Parameter	Estimate ± SE	*p*-value	AUC^a^	$\:{\mathrm{x}}_{1}$value for *P* = 50%
Intercept (β_0_)	-11.986 ± 8.593	0.163	0.608	135.13
Withers height (β_1_)	0.089 ± 0.072	0.215		
Intercept (β_0_)	-18.002 ± 7.632	0.018	0.729	45.30
Rump length (β_1_)	0.397 ± 0.181	0.028		
Intercept (β_0_)	-15.08 ± 5.844	0.010	0.738	40.87
Ilium width (β_1_)	0.369 ± 0.155	0.017		
Intercept (β_0_)	-9.502 ± 4.265	0.026	0.731	28.47
Ischium width (β_1_)	0.334 ± 0.173	0.054		
Intercept (β_0_)	-9.011 ± 3.353	0.007	0.750	1506.91
Rump area (β_1_)	0.006 ± 0.003	0.020		
Intercept (β_0_)	-6.849 ± 2.789	0.014	0.708	339.06
Estimated weight (β_1_)	0.020 ± 0.010	0.044		

^a^AUC: area under ROC curve; value for *P* = 50%: This value shows the point at which the probability of success (ovulation) reaches 50%

In multivariate logistic regression, twenty models were pre-selected and ranked by Score criterion (Suppl Table 2). A model including GG, BCS, withers height, rump area, and estimated weight was selected for multivariate analysis.

According to AICc values, rump area and withers height were the most influential variables; nevertheless, only rump area showed tendency (*p* < 0.06) to affect probability of ovulation (Table 3). Although it has a marginal effect, rump area positively affected probability of ovulation with an OR = 4.055 per 100 cm^2^, that is, more than quadrupling the odds per 100 cm^2^ increase.

**Table 3:** Model fit parameters and analysis of maximum likelihood of the multivariate logistic regression to evaluate probability of ovulation in Girolando heifers

Parameter	Model Fit		Analysis of Maximum likelihood	
	AICc	Delta^a^	Estimate ± SE	Pr > ChiSq
Intercept	62.048	n.a.	11.666 ± 16.616	0.483
Rump Area (cm^2^)	57.729	-4.319	0.014 ± 0.007	0.055
Withers Height (cm)	55.380	-2.349	-0.291 ± 0.191	0.127
BCS (Score)	56.396	+ 1.016	1.380 ± 1.389	0.320
GG	58.173	+ 1.777	0.202 ± 0.424	0.634
Estimated weight (Kg)	60.172	+ 1.999	0.001 ± 0.028	0.973

^a^In the forward selection, variables were added to the logistic model in descending order (starting with the “Intercept” model and ending with adding “Estimated weight”). In each step, the AICc after adding a variable was compared with the AICc of the previous step; n.a. not applicable. BCS: body condition score; GG: genetic group

Of the 60 heifers enrolled in the study, 12 (20%) reached ovulation during the experimental period. To investigate the association between time to ovulation and 365-days-adjusted morphometric traits, a Cox proportional hazards model was employed. Initially, a full model including all potential predictors was fitted (Suppl Table 3). The proportional hazards assumption was assessed using Schoenfeld residuals, and no significant violations were detected (Suppl Table 4). Multicollinearity and Pearson correlations among predictors are provided in the Suppl Table 5.

After evaluating 20 candidate models (Suppl Table 6), the final Cox model included genetic group (GG), body condition score (BCS), and rump area, with the area trait modeled using a penalized spline. The final model satisfied the proportional hazards assumption (Suppl Table 7).

**Fig. 3:**
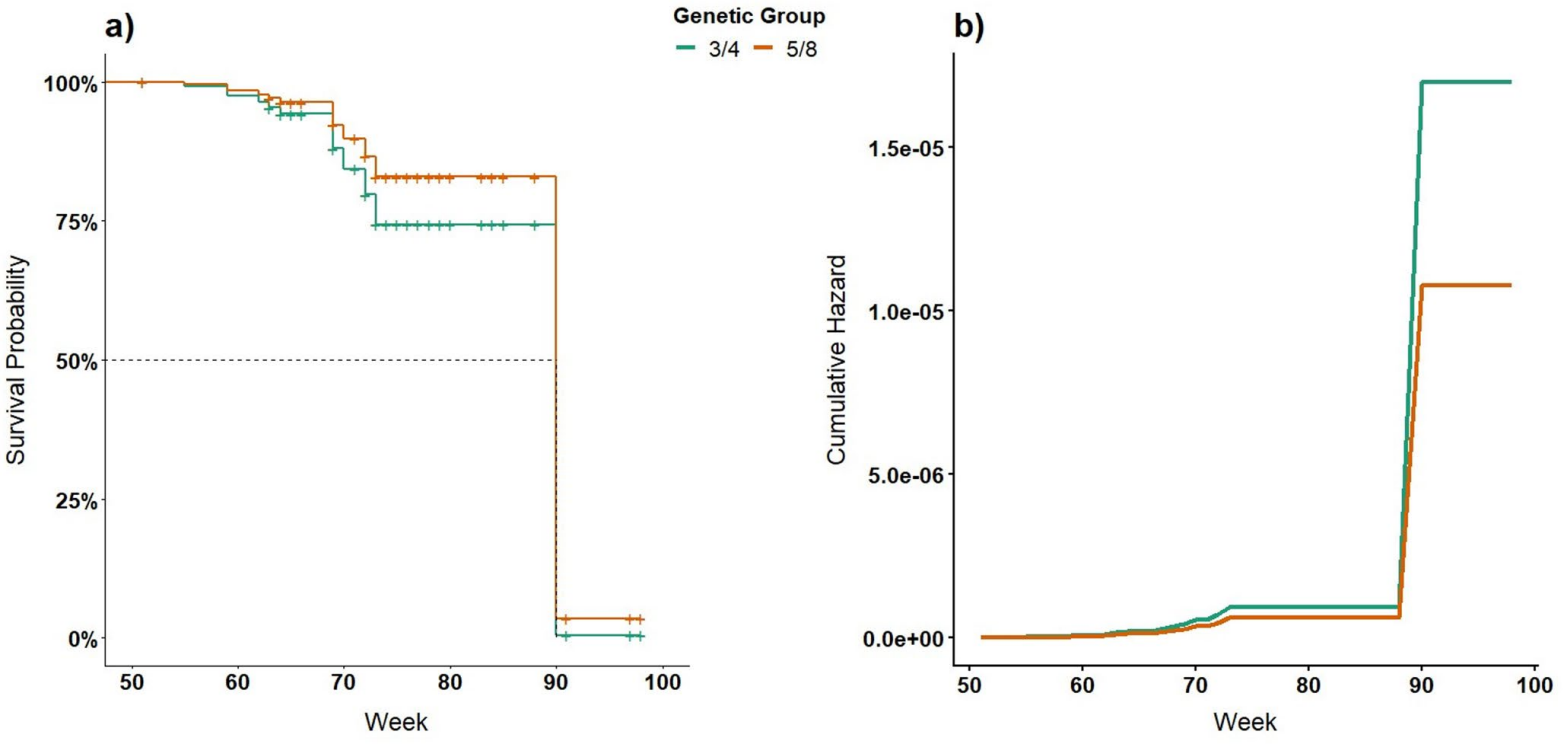
Survival (**a**) and Cumulative Hazard (**b**) curves for time-to-ovulation based on Cox model in Girolando heifers, according to genetic group

Genetic group and BCS were not significantly associated with time to ovulation (Table 4). In the adjusted model, ovulation events were observed around the 70th week, with a more pronounced increase near the 90th week, reaching the median (50% ovulation; Fig. 3). It is noteworthy that in the raw, unadjusted data, the median was not achieved for either genetic group.

Importantly, the 365-days-adjusted rump area showed a significant linear association with time-to-ovulation (Table 4). The linear component of the penalized spline for rump area was statistically significant (*p* < 0.01), indicating that larger rump areas were associated with a higher hazard of ovulation, i.e., shorter time to ovulation. The non-linear component showed marginal effect (*p* < 0.10), suggesting limited evidence of deviation from linearity.

**Fig. 4:**
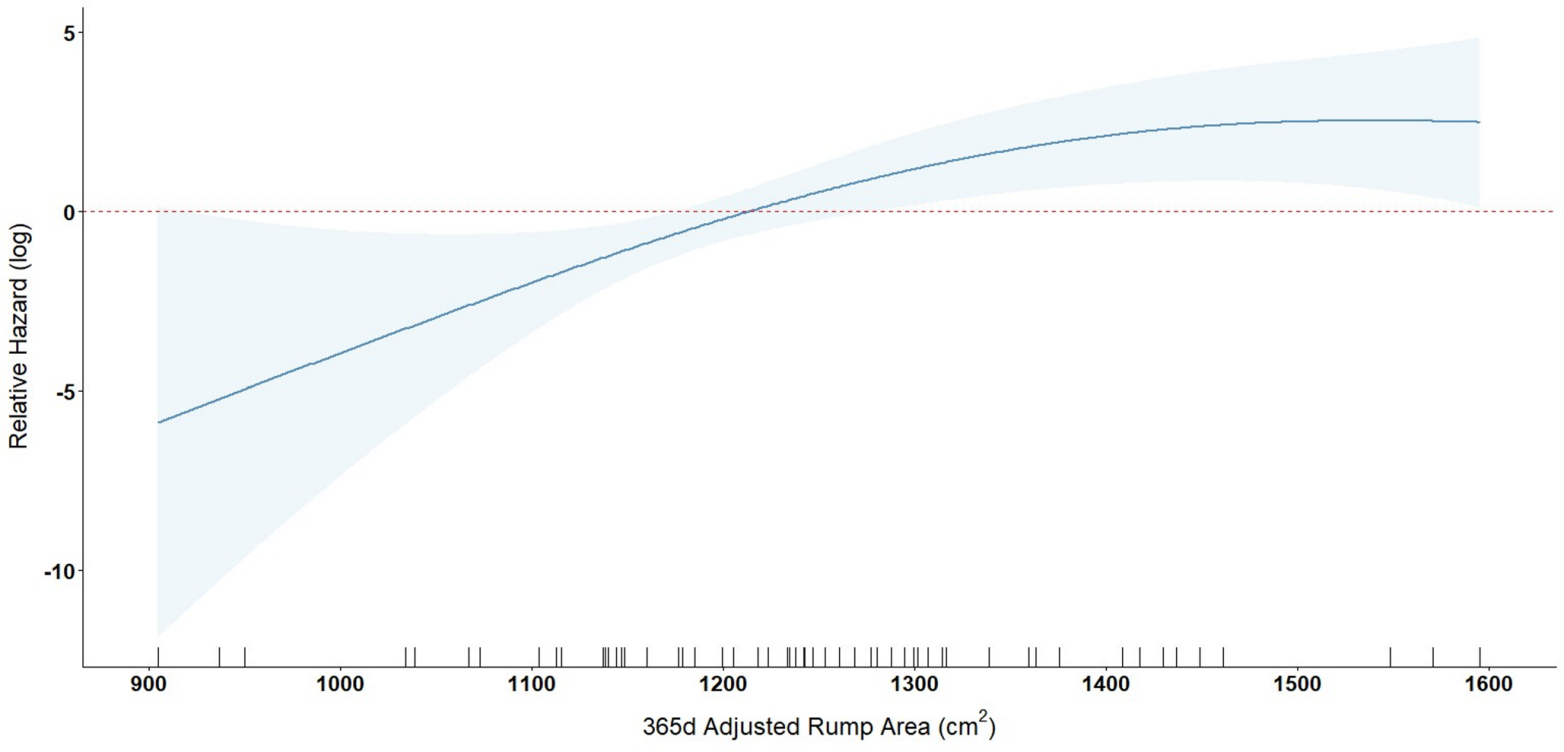
Log-relative hazard of ovulation as a function of rump area (cm²), adjusted to 365 days of age. The solid line represents the estimated log-relative hazard from a Cox proportional hazards model using a penalized spline (df = 2), centered at the mean rump area. Shaded areas represent 95% confidence intervals. The dashed line indicates the reference value (log-hazard = 0), corresponding to the mean rump area. Rug ticks on the x-axis show the distribution of observed rump area values

The estimated hazard ratio for the linear component of rump area was 1.008 (95% CI: 1.002–1.015), indicating that each additional 1 cm² in rump area increased the hazard of ovulation by 0.8%. This suggests that heifers with larger 365-day-adjusted rump areas tended to ovulate earlier (Fig. 4), and the relationship appeared linear without significant curvature.

**Table 4:** Final Cox model to evaluate the association between predictors and time-to-ovulation in Girolando heifers

Parameter	Coefficient		*p*-value	Hazard Ratio (HR)		
				HR (e^(coefficient)^)	95% LCI	95% UCI
GG (3/4)	0.457	0.500		1.580	0.414	6.021
BCS	1.444	0.150		4.237	0.590	30.043
Spline (Rump area) – linear	0.008	0.009		1.008	1.002	1.015
Spline (Rump area) – non-linear	-	0.094		-	-	-

GG, genetic group (5/8 GG was the reference). BCS, body condition score. The effect of rump area on the hazard of ovulation was modeled using a penalized spline with 2 degrees of freedom

The ANOVA results for morphometric traits between 3/4 and 5/8 GG at the time of ovulation are available on Table 5. Rump area and Ischium width had tendency to be larger in 3/4 GG heifers (*p* < 0.06).

**Table 5:** Values of morphometric traits and age of 3/4 and 5/8 Girolando heifers at the time of ovulation (*N* = 12)

Trait	3/4 GG	5/8 GG	*p*-value
Proportion	27.8% (5/18)	16.7% (7/42)	-
Withers height (cm)	129.00 ± 1.05	125.43 ± 2.03	0.198
Rump length (cm)	45.98 ± 0.24	45.19 ± 0.45	0.197
Ilium width (cm)	42.40 ± 0.43	41.67 ± 0.40	0.250
Ischium width (cm)	29.14 ± 0.62	26.27 ± 1.01	0.054
Rump area (cm^2^)	1627.14 ± 16.83	1513.32 ± 41.47	0.052
Estimated Weight (kg)	337.60 ± 7.19	340.00 ± 10.11	0.863
Age (days)	452.80 ± 20.18	481.14 ± 28.99	0.415

GG: genetic group

## Discussion

The use of practical and empirical assessments, such as body weight and body condition score (BCS), has become more common (Martin et al. 2008) and efficient (Dickinson et al. 2019) than relying solely on animals’ chronological age for evaluating reproductive readiness. More recently, morphometric traits related to pelvic bone structure have emerged as potential morphological markers (Credille et al. 2023). The present study extends previous findings by jointly evaluating traditional indicators (body weight and BCS) and detailed pelvic morphometric measurements as predictors of ovulation in Girolando heifers, specifically comparing animals from the 3/4 and 5/8 genetic groups. By integrating linear mixed models, logistic regression, and survival analysis, this study provides a quantitative framework to assess how morphometric variation at 365 days of age relates not only to the probability of ovulation, but also to the timing of its occurrence These indicators, when validated in more comprehensive studies, can be consolidated as valuable tools for the selection of reproductive traits in the Girolando breed.

In this study, the body growth was not continuous and linear for the heifers, which can be attributed to environmental variations in the conditions under which they were raised. Despite apparent stability in the general environment, factors such as climate fluctuations (Tsiamadis et al. 2023), forage quality (Greenland et al. 2023), management system (Roberts et al. 2017), and sanitary conditions may change over time (Alfieri et al. 2019). Furthermore, the type and quality of diets offered during rearing play a crucial role in growth and development (Garza et al. 2023). Studies suggest that breeding dairy heifers for precocious calving (22–24 months) is feasible if adequate body size and organ development are achieved (Fantuz et al. 2024).

In this study, there was insufficient evidence to support a statistically significant difference between genetic groups. However, the higher proportion of pubescent heifers in GG 3/4 may be attributed to the higher proportion of Holstein blood (75% versus 62.5%, on average), which favors early sexual maturity but may reduce resistance to heat stress (Teixeira et al. 2019). These findings reinforce the importance of considering genetic and environmental factors, such as heterosis and feed management, to better understand puberty and sexual maturity in heifers.

Currently, many studies rely on data from large farms or experimental facilities; however, few address the reality of small-scale producers, who represent a substantial portion of Brazilian livestock farming. Thus, the imbalance in the number of heifers per GG observed in this study, as well as the age differences between the animals, required extensive statistical adjustments, as evidenced in other studies (Pereira et al. 2017; Fantuz et al. 2024).

The differences in experimental group sizes reflect the heterogeneous realities on commercial farms. Sexual maturity varies according to genetics and environmental factors, such as nutrition, which can account for up to 20% of the observed variability (Hileman et al. 2020). For replacement dairy heifers, advances in genetics and nutrition increase milk production, promoting greater body growth and weight at first calving (Kusaka et al. 2023). However, many producers invest in high-quality genetics but allocate heifers to less productive areas without adequate shaded, compromising their development (Cardoso et al. 2021).

On the farm where this study was conducted, heifers were supplemented with corn silage, soybean meal, cornmeal and mineral salt, which favored the achievement of ideal weight and size for insemination. The literature highlights that appropriate feeding during rearing is essential to anticipate puberty, improve reproductive efficiency and increase the economic returns (Amstalden et al. 2011). However, heifers that begin their productive life later (after 28 months) show substantial reductions in lifetime milk production, fewer calvings, and higher culling rates due to low productivity and udder diseases (Nilforooshan and Edriss 2004).

Despite the recent focus on reproductive longevity in cows (Heise et al. 2016; Bot Steffl et al. 2024), the actual productive lifespan of animals has shortened, possibly due to inadequate management and pressure for high productivity (Britt et al. 2021). According to the results obtained in this study, the minimum age for Girolando heifers, regardless of genetic composition, to reach puberty is 55 weeks (approximately 13 months). However, due to logistical and operational constraints inherent to the routine of the commercial farm, it was not possible to monitor the exact onset of puberty in all animals, resulting in partially censored data for this variable.

The mean age of puberty observed for Girolando 3/4 and 5/8 heifers was 452.8 ± 20.18 and 481.14 ± 28.99 days, respectively. These values ​​differ from those reported by Fonseca et al. (2020), who found earlier average ages in F1 Holstein × Gir heifers. This difference can be attributed to heterosis, which favors greater precocity and productivity in F1 crosses (Silva et al. 2011; Santana et al. 2014).

The ideal time for insemination of heifers depends not only on age but also on body weight and BCS, which directly reflect nutritional and hormonal status (Perry 2016). Unlike BCS, body weight is related to milk production in primiparous cows but may negatively affect conception rate (Lauber and Fricke 2023). In our study, in addition to body weight, rump area significantly influenced the probability of ovulation. This parameter reflects muscle development and fat deposition, which are essential for hormonal balance and reproductive efficiency (Cardoso et al. 2018; Nogalski et al. 2024).

Although no significant difference was observed between the genetic groups in average body weight at puberty, inadequate nutritional management and dry season result in significant weight losses, compromising reproductive development. Abrupt changes in diet can disrupt the physiological system of heifers, initially affecting body weight and subsequently hormonal balance (Senger 2003), which directly impacts body condition, a fundamental factor for sexual maturation and reproductive efficiency (Ferreira 2010). Therefore, ensuring consistent and adequate food management is essential to avoid losses in reproductive performance, corroborating the importance of body indicators, such as weight and rump area, for the assessment of reproductive fitness.

The effect of age, assessed by regression analysis, was significant for all body measurements analyzed in both genetic groups, which was expected considering that young animals are still in physiological and morphological development (Fantuz et al. 2024). Furthermore, most body measurements were similar between GG, excepting withers height and ischium width that were slightly larger in 3/4 GG. Similarities in body measurements in locally adapted Holstein and Holstein × Gyr dairy heifers were reported by Abreu et al. (2022).

Among the evaluated traits, rump area showed marginal association with ovulation probability. This result suggests that factors related to overall body development exert a stronger influence on ovulation than genetic differences between GG per se. Moreover, rump area emerged as the most influential factor for puberty onset according to the multivariate Cox model, representing the primary variable associated with time-to-ovulation. Machado et al. (2024) reported moderate to high genetic correlations between ilium width, rump area, and hip height and reproductive traits in Gyr cattle, including number of total and viable oocytes, and number of embryos. These findings emphasize the importance of evaluating body traits as indirect indicators of reproductive potential.

These pelvic traits have a promising role in the selection and management of heifers since body measurements such as ileum width and rump area are closely linked to reproductive capacity (Bila et al. 2021). A wider pelvis and a greater distance between the ischial tuberosities not only indicate a more adequate physical development to support pregnancy, but also facilitate birth, reflecting ideal reproductive maturity (Holm et al. 2016). It is worth noting that these measurements can help predict the animal’s response to the metabolic and physical demands of pregnancy and lactation, which is particularly relevant in dairy breeds such as Girolando. Validation of these traits could benefit genetic improvement programs (Stafuzza et al. 2017) and reduce reproductive losses, enabling more informed management decisions on dairy farms (Atzori et al. 2023).

The rump area stands out as an important variable, being one of the main factors associated with incidence of dystocia (Nogalski and Barański 2023). Variables such as rump area, rump height and other linear body measurements contribute to safe pregnancies and successful parturition (Bila et al. 2021). Our results indicate that rump measurements are more strongly associated with puberty than variables such as BCS and body weight suggesting that these measures represent useful tools for predicting age at puberty. This may reduce unprofitable investments in less promising animals (Bach 2011).

Survival analysis revealed valuable information about the time to puberty in Girolando heifers. The survival rate, indicating the probability of heifers not reaching puberty, began to decrease from the 55th week of age, indicating that puberty occurs mostly after this period. This information may allow for the optimization of nutritional management, aiming to increase pre-weaning weight, reduce age at puberty, and anticipate sexual maturity (Brunes et al. 2022). Although the 3/4 and 5/8 genetic groups showed distinct patterns in the survival curves, no significant differences were found between them. In contrast, Teixeira et al. (2019) also investigated these same genetic groups and found significant differences in male reproductive traits, such as semen quality and thermoregulation, highlighting potential sex-specific effects of genetic composition.

In conclusion, both rump area and ischium width showed marginal differences between 3/4 and 5/8 Girolando heifers at the time of first ovulation, suggesting that genetic composition might slightly influence body traits associated with reproductive fitness. Among these traits, rump area emerged as the most influential predictor to ovulation probability and time-to-ovulation, reinforcing its potential as a key phenotypic indicator for reproductive management in Girolando females, although studies with larger sample sizes are still required to validate these results. These findings contribute to a better understanding of reproductive development in this composite breed and offer valuable support for improving selection strategies and productivity, especially in small-scale dairy systems.

## Supplementary Information

Below is the link to the electronic supplementary material.

Supplementary Material 1

## Data Availability

The datasets generated and analyzed during this study are available from the corresponding author on reasonable request.
