# Supplementary material for: Morphological indicators of puberty in 3/4 and 5/8 Girolando heifers
Source: Trop Anim Health Prod. 2026 May 20;58(5):278. doi: 10.1007/s11250-026-05078-7 (PMC13190439; doi:10.1007/s11250-026-05078-7)
Supplement: Supplementary file 1 — Supplementary Material 1 [file 11250_2026_5078_MOESM1_ESM.docx]

Suppl Table 1. Descriptive statistics (averages ± standard deviations) of body traits by age (in months) for 3/4 and 5/8 Girolando heifers

| Age  (months) | GG | WH (cm) | RL (cm) | ILW(cm) | ISW (cm) | RA (cm^2^) | EW (kg) |
| --- | --- | --- | --- | --- | --- | --- | --- |
| 7 | 3/4 | 112 ± 2.6 | 38.1 ± 1.2 | 31.5 ± 1.3 | 23.7 ± 0.4 | 1048.8 ± 64 | 199.7 ± 17.9 |
|  | 5/8 | 108 ± 4.4 | 37.3 ± 1.6 | 31.1 ± 1.9 | 21.4 ± 1.6 | 972.7 ± 90.6 | 198.8 ± 24.1 |
| 8 | 3/4 | 114.2 ± 2.8 | 37.8 ± 2.4 | 33.9 ± 1.7 | 23.5 ± 1.8 | 1073.3 ± 90.6 | 213.6 ± 15.6 |
|  | 5/8 | 109.9 ± 4.3 | 38.2 ± 1.8 | 32.1 ± 1.7 | 21.7 ± 1.5 | 1021.1 ± 101.2 | 205.1 ± 14.2 |
| 9 | 3/4 | 115.2 ± 3.7 | 39.6 ± 1.5 | 34.6 ± 2 | 23.8 ± 1.5 | 1147 ± 96.3 | 235.1 ± 23.2 |
|  | 5/8 | 112 ± 3.4 | 38.6 ± 1.7 | 32.9 ± 1.5 | 22.7 ± 1.4 | 1063.8 ± 86.4 | 213 ± 18 |
| 10 | 3/4 | 118.6 ± 3.8 | 40.4 ± 1.8 | 35.5 ± 2.8 | 23.7 ± 3.1 | 1186.3 ± 160.5 | 252.7 ± 27.9 |
|  | 5/8 | 114.3 ± 3.6 | 39.3 ± 1.7 | 34.1 ± 1.9 | 21.8 ± 2.3 | 1085.6 ± 90.3 | 231.1 ± 21.6 |
| 11 | 3/4 | 120.2 ± 4.8 | 41.3 ± 2 | 36.8 ± 2.5 | 23.6 ± 3.3 | 1232 ± 148.8 | 264.1 ± 34.1 |
|  | 5/8 | 116.3 ± 4.6 | 40.2 ± 2.1 | 35.1 ± 2.1 | 22.7 ± 2.3 | 1149.4 ± 131 | 244.3 ± 27.4 |
| 12 | 3/4 | 123.2 ± 5.3 | 43.2 ± 2.2 | 38.4 ± 2.8 | 26.3 ± 3 | 1387.5 ± 175 | 292.3 ± 33.8 |
|  | 5/8 | 118.3 ± 5.3 | 41.5 ± 1.9 | 36.4 ± 2.2 | 23.9 ± 2.1 | 1237.7 ± 130.1 | 255.7 ± 29.9 |
| 13 | 3/4 | 123.3 ± 5.3 | 43 ± 2.8 | 38.7 ± 3.1 | 26.4 ± 2.7 | 1392.8 ± 200.5 | 293 ± 48 |
|  | 5/8 | 119.6 ± 5.1 | 41.9 ± 2.2 | 37.2 ± 2.3 | 23.9 ± 2.8 | 1267.5 ± 148.1 | 267.1 ± 34.4 |
| 14 | 3/4 | 126.7 ± 5.1 | 44.4 ± 3 | 40.7 ± 2.9 | 27 ± 3.6 | 1492 ± 215.8 | 323.9 ± 46.1 |
|  | 5/8 | 122.5 ± 5.1 | 43 ± 2.4 | 38.4 ± 2.8 | 24.4 ± 3.2 | 1336.4 ± 179.6 | 292.1 ± 42.5 |
| 15 | 3/4 | 127.2 ± 5.2 | 44.7 ± 2.6 | 40.6 ± 3 | 27.5 ± 3.3 | 1509 ± 204.8 | 328.8 ± 49.6 |
|  | 5/8 | 124.1 ± 5.1 | 44 ± 2.3 | 39.5 ± 2.3 | 26.2 ± 2 | 1431.5 ± 153.2 | 310.2 ± 38 |
| 16 | 3/4 | 127 ± 4 | 44.1 ± 2.3 | 40.5 ± 3.4 | 27.5 ± 3 | 1489.1 ± 189.4 | 311.2 ± 43.3 |
|  | 5/8 | 124.3 ± 4 | 44.2 ± 1.7 | 39.6 ± 2.1 | 26.6 ± 1.7 | 1448.2 ± 118.7 | 312.4 ± 36.3 |
| 17 | 3/4 | 126.5 ± 4.5 | 44.4 ± 2.5 | 40.3 ± 2.5 | 27.8 ± 1.8 | 1499.2 ± 163.6 | 321.2 ± 38.9 |
|  | 5/8 | 124.9 ± 4.6 | 44.4 ± 1.7 | 39.8 ± 2.9 | 26.4 ± 1.8 | 1455.7 ± 118.3 | 312.9 ± 30.7 |
| 18 | 3/4 | 128.8 ± 6 | 44.8 ± 2.7 | 41.1 ± 3.1 | 29.6 ± 2.9 | 1577.9 ± 225.8 | 321.8 ± 35.2 |
|  | 5/8 | 125.3 ± 3.7 | 44.8 ± 1.4 | 40.6 ± 1.4 | 26.5 ± 1.9 | 1484.8 ± 101.4 | 319.8 ± 29.3 |
| 19 | 3/4 | 125.8 ± 3.6 | 43.4 ± 2.4 | 39.7 ± 1.8 | 28.1 ± 1.2 | 1458.9 ± 144.5 | 310.8 ± 26.9 |
|  | 5/8 | 125.3 ± 3 | 44.8 ± 1.1 | 41.1 ± 2.3 | 26.7 ± 2.3 | 1499 ± 123.8 | 332 ± 24.7 |
| 20 | 3/4 | 127.7 ± 4.9 | 44.9 ± 2.9 | 41.1 ± 1.7 | 28.4 ± 0.2 | 1545.8 ± 137 | 337.7 ± 20.1 |
|  | 5/8 | 126 ± 1.9 | 44.8 ± 1.3 | 40.6 ± 1.4 | 25.8 ± 2.2 | 1469 ± 104 | 312.6 ± 27 |

GG: genetic group; WH: withers height; RL: rump length; ILW: ilium width; ISW: Ischium width; RA: Rump area; EW: Estimated weight


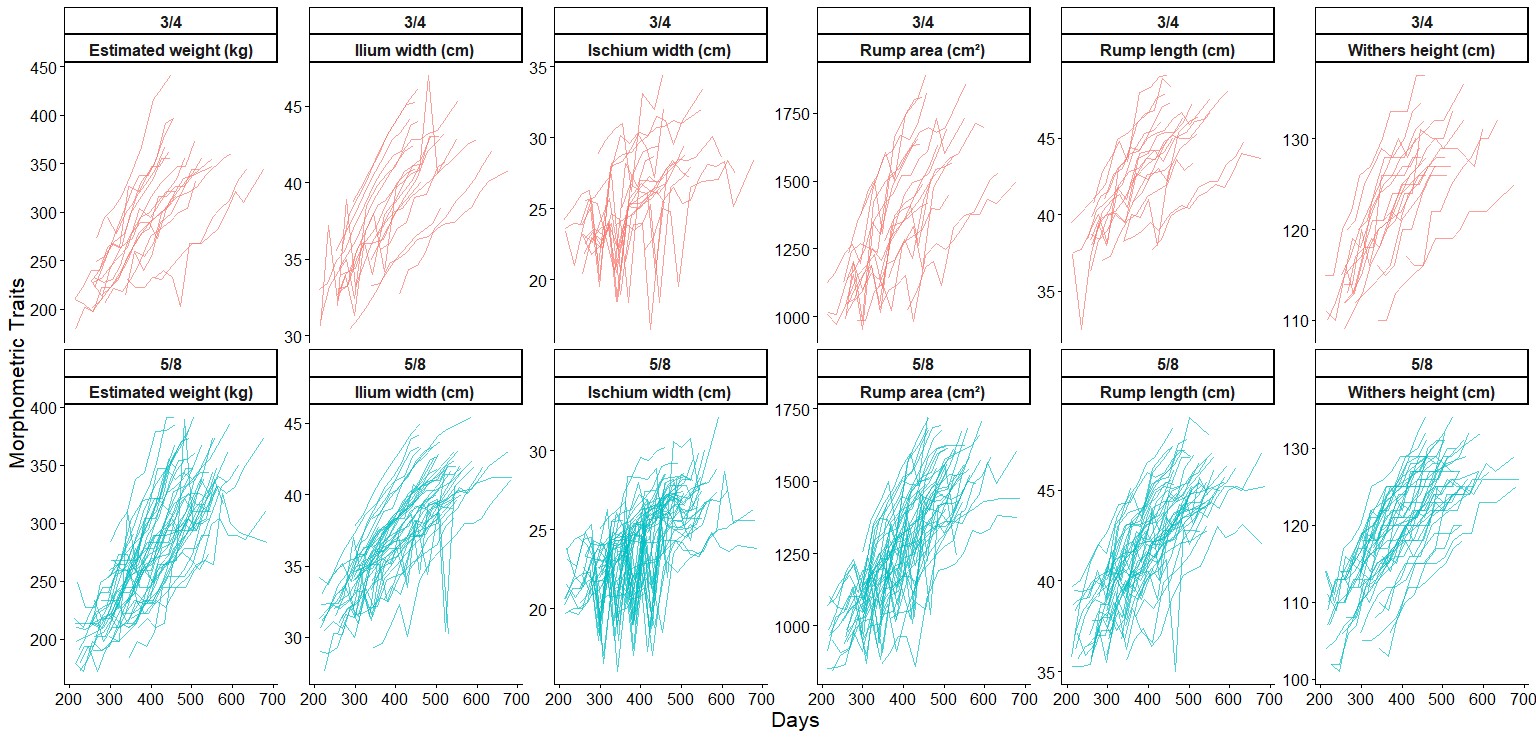


Suppl Fig 1. Observed morphometric traits values over time (in days) in each genetic group.

Suppl Fig 2. Pearson correlation coefficients between age and morphometric traits for 3/4 and 5/8 Girolando heifers.

Suppl Table 2. Chi-square Score of the candidate models for multivariate logistic regression.

| **GG** | **BCS** | **Withers** | **Area** | **Weight** | **Ilium** | **Rump** | **Ischium** | **Score** |
| --- | --- | --- | --- | --- | --- | --- | --- | --- |
| **✓** | **✓** | **✓** | **✓** | **✓** |  |  |  | 11.388 |
| **✓** | **✓** | **✓** | **✓** |  |  |  |  | 11.372 |
| **✓** | **✓** | **✓** |  | **✓** |  | **✓** |  | 10.551 |
| **✓** | **✓** | **✓** |  |  |  | **✓** |  | 10.550 |
| **✓** | **✓** | **✓** |  | **✓** | **✓** |  |  | 9.508 |
| **✓** | **✓** | **✓** |  |  | **✓** |  |  | 9.320 |
| **✓** | **✓** |  | **✓** | **✓** |  |  |  | 9.079 |
| **✓** | **✓** |  |  | **✓** |  | **✓** |  | 8.546 |
| **✓** | **✓** |  | **✓** |  |  |  |  | 8.443 |
| **✓** | **✓** | **✓** |  | **✓** |  |  | **✓** | 8.365 |
| **✓** | **✓** |  |  | **✓** | **✓** |  |  | 8.357 |
| **✓** | **✓** |  |  |  | **✓** |  |  | 8.256 |
| **✓** | **✓** |  |  |  |  | **✓** |  | 8.171 |
| **✓** | **✓** |  |  | **✓** |  |  | **✓** | 7.474 |
| **✓** | **✓** | **✓** |  |  |  |  | **✓** | 7.431 |
| **✓** | **✓** |  |  |  |  |  | **✓** | 7.331 |
| **✓** | **✓** | **✓** |  | **✓** |  |  |  | 7.245 |
| **✓** | **✓** |  |  | **✓** |  |  |  | 6.825 |
| **✓** | **✓** | **✓** |  |  |  |  |  | 5.813 |
| **✓** | **✓** |  |  |  |  |  |  | 5.005 |

Suppl Table 3. Initial Cox Model Results

| Parameter | Coefficient | Pr(>\|z\|) | Hazard Ratio (HR) | | |
| --- | --- | --- | --- | --- | --- |
|  |  |  | HR  (e^(coefficient)^) | 95% LCI | 95% UCI |
| GG (3/4) | 0.060 | 0.941 | 1.062 | 0.216 | 5.219 |
| Withers height | -0.408 | 0.020 | 0.665 | 0.472 | 0.937 |
| BCS | 0.851 | 0.511 | 2.343 | 0.185 | 29.716 |
| Rump length | 4.146 | 0.103 | 63.186 | 0.435 | 9172.21 |
| Ilium width | 2.139 | 0.120 | 8.490 | 0.574 | 125.509 |
| Ischium width | 2.898 | 0.134 | 18.144 | 0.411 | 801.144 |
| Rump area | -0.108 | 0.161 | 0.897 | 0.772 | 1.044 |
| Weight | 0.033 | 0.232 | 1.034 | 0.979 | 1.091 |

GG, genetic group.

Suppl Table 4. Proportional Hazards Assumption of the initial model.

| Variable | Chi-square | df | p-value |
| --- | --- | --- | --- |
| GG | 0.11156 | 1 | 0.74 |
| Withers height | 0.21119 | 1 | 0.65 |
| BCS | 0.82944 | 1 | 0.36 |
| Rump length | 0.16589 | 1 | 0.68 |
| Ilium width | 0.00706 | 1 | 0.93 |
| Ischium width | 0.08331 | 1 | 0.77 |
| Rump area | 0.09719 | 1 | 0.76 |
| Weight | 0.40108 | 1 | 0.53 |
| Global | 8.46702 | 8 | 0.39 |

GG, genetic group.

Suppl Table 5. Multicollinearity evaluation among potential predictors.

| *Pearson correlation* | | | | | | | | | | | | | |
| --- | --- | --- | --- | --- | --- | --- | --- | --- | --- | --- | --- | --- | --- |
|  | | Withers | | Rump | | Ilium | | Ischium | | Area | | Weight | |
| Withers | | 1.0000 | | 0.8831 | | 0.8137 | | 0.7614 | | 0.8910 | | 0.8674 | |
| Rump | |  | | 1.0000 | | 0.8709 | | 0.6999 | | 0.9452 | | 0.9072 | |
| Ilium | |  | |  | | 1.0000 | | 0.7463 | | 0.9396 | | 0.8687 | |
| Ischium | |  | |  | |  | | 1.0000 | | 0.8732 | | 0.7383 | |
| Area | |  | |  | |  | |  | | 1.0000 | | 0.9191 | |
| Weight | |  | |  | |  | |  | |  | | 1.0000 | |
|  | |  | |  | |  | |  | |  | |  | |
| *VIF* | | | | | | | | | | | | | |
| GG | Withers | | BCS | | Rump | | Ilium | | Ischium | | Area | | Weight |
| 1.6501 | 7.0001 | | 1.0971 | | 187.5700 | | 72.6478 | | 171.1205 | | 889.9089 | | 7.6217 |

Suppl Table 6. Model selection table.

| Intercept | Area | BCS | GG | Ilium | Ischium | rump | Weight | Withers | AICc | Delta | Weight |
| --- | --- | --- | --- | --- | --- | --- | --- | --- | --- | --- | --- |
| + | 0.0088 | 1.510 | + |  |  |  |  |  | 77.8 | 0.00 | 0.249 |
| + |  | 1.419 | + | 0.5184 |  |  |  |  | 78.4 | 0.59 | 0.186 |
| + |  | 1.490 | + |  |  | 0.5332 |  |  | 79.5 | 1.64 | 0.110 |
| + |  | 1.731 | + |  |  |  | 0.0316 |  | 80.4 | 2.54 | 0.070 |
| + | 0.0143 | 1.214 | + |  |  |  |  | -0.1912 | 80.8 | 2.99 | 0.056 |
| + |  | 1.360 | + | 0.3553 |  | 0.2561 |  |  | 82.1 | 4.29 | 0.029 |
| + | 0.0057 | 1.430 | + | 0.2132 |  |  |  |  | 82.2 | 4.39 | 0.028 |
| + | 0.0104 | 1.437 | + |  | -0.1252 |  |  |  | 82.3 | 4.50 | 0.026 |
| + | 0.0075 | 1.484 | + |  |  | 0.1053 |  |  | 82.5 | 4.63 | 0.025 |
| + | 0.0079 | 1.497 | + |  |  |  | 0.0048 |  | 82.5 | 4.65 | 0.024 |
| + |  | 1.385 | + | 0.4040 |  |  | 0.0105 |  | 82.7 | 4.89 | 0.022 |
| + |  | 1.443 | + | 0.4677 | 0.0798 |  |  |  | 83.0 | 5.16 | 0.019 |
| + |  | 1.381 | + | 0.5672 |  |  |  | -0.0357 | 83.0 | 5.19 | 0.019 |
| + |  | 1.567 | + |  | 0.2123 | 0.4379 |  |  | 83.0 | 5.19 | 0.019 |
| + |  | 2.105 | + |  | 0.4172 |  |  |  | 83.1 | 5.29 | 0.018 |
| + |  | 1.215 | + |  |  | 0.8209 |  | -0.1535 | 83.3 | 5.42 | 0.017 |
| + |  | 1.503 | + |  |  | 0.3786 | 0.0119 |  | 83.8 | 5.95 | 0.013 |
| + |  | 1.747 | + |  | 0.2041 |  | 0.0256 |  | 84.0 | 6.17 | 0.011 |
| + |  | 2.126 | + |  |  |  |  | 0.1812 | 84.2 | 6.40 | 0.010 |
| + |  | 1.717 | + |  |  |  | 0.0414 | -0.0870 | 84.8 | 6.94 | 0.008 |

Suppl Table 7. Proportional Hazards Assumption of the final model

| Variable | Chi-square | df | p-value |
| --- | --- | --- | --- |
| GG | 0.0105 | 0.98 | 0.91 |
| BCS | 0.8606 | 0.97 | 0.34 |
| Rump area | 0.1949 | 1.99 | 0.91 |
| Global | 1.1310 | 3.94 | 0.88 |

GG, genetic group.
